# Mathematical Modeling of Transmission Dynamics and Optimal Control of Vaccination and Treatment for Hepatitis B Virus

**DOI:** 10.1155/2014/475451

**Published:** 2014-04-09

**Authors:** Ali Vahidian Kamyad, Reza Akbari, Ali Akbar Heydari, Aghileh Heydari

**Affiliations:** ^1^Department of Mathematics Sciences, Ferdowsi University of Mashhad, Mashhad, Iran; ^2^Department of Mathematical Sciences, Payame Noor University, Iran; ^3^Research Center for Infection Control and Hand Hygiene, Mashhad University of Medical Sciences, Mashhad, Iran; ^4^Payame Noor University, Khorasan Razavi, Mashhad, Iran

## Abstract

Hepatitis B virus (HBV) infection is a worldwide public health problem. In this paper, we study the dynamics of hepatitis B virus (HBV) infection which can be controlled by vaccination as well as treatment. Initially we consider constant controls for both vaccination and treatment. In the constant controls case, by determining the basic reproduction number, we study the existence and stability of the disease-free and endemic steady-state solutions of the model. Next, we take the controls as time and formulate the appropriate optimal control problem and obtain the optimal control strategy to minimize both the number of infectious humans and the associated costs. Finally at the end numerical simulation results show that optimal combination of vaccination and treatment is the most effective way to control hepatitis B virus infection.

## 1. Introduction

Hepatitis B is a potentially life-threatening liver infection caused by the hepatitis B virus. It is a major global health problem. It can cause chronic liver disease and chronic infection and puts people at high risk of death from cirrhosis of the liver and liver cancer [1]. Infections of hepatitis B occur only if the virus is able to enter the blood stream and reach the liver. Once in the liver, the virus reproduces and releases large numbers of new viruses into the blood stream [2].

This infection has two possible phases: (1) acute and (2) chronic. Acute hepatitis B infection lasts less than six months. If the disease is acute, your immune system is usually able to clear the virus from your body, and you should recover completely within a few months. Most people who acquire hepatitis B as adults have an acute infection. Chronic hepatitis B infection lasts six months or longer. Most infants infected with HBV at birth and many children infected between 1 and 6 years of age become chronically infected [1]. About two-thirds of people with chronic HBV infection are chronic carriers. These people do not develop symptoms, even though they harbor the virus and can transmit it to other people. The remaining one-third develop active hepatitis, a disease of the liver that can be very serious. More than 240 million people have chronic liver infections. About 600 000 people die every year due to the acute or chronic consequences of hepatitis B [1].

Transmission of hepatitis B virus results from exposure to infectious blood or body fluids containing blood. Possible forms of transmission include sexual contact, blood transfusions and transfusion with other human blood products (horizontal transmission), and possibly from mother to child during childbirth (vertical transmission) [3]. The most important influence on the probability of developing carriage after infection is age [4]. Children less than 6 years of age who become infected with the hepatitis B virus are the most likely to develop chronic infections:

 80–90% of infants infected during the first year of life develop chronic infections;
 30–50% of children infected before the age of 6 years develop chronic infections.
 In adults:
   <5% of otherwise healthy adults who are infected will develop chronic infection;
   15–25% of adults who become chronically infected during childhood die from hepatitis B related liver cancer or cirrhosis [1, 5].

Patients with chronic carrier often have no history of acute illness but may develop cirrhosis (liver scarring) that can lead to liver failure and may also develop liver cancer [5]. A small portion (1–6%) of chronic carries clear the virus naturally [5]. Someone who is infected with hepatitis B may have symptoms similar to those caused by other viral infections. However, many people infected with hepatitis B do not have any symptoms until when more serious side effects such as liver damage can occur. Someone who has been exposed to hepatitis B may have signs 2 to 5 months later. Some people with hepatitis B do not notice warning signs until they become very intense. Some have few or no symptoms, but even someone who does not notice any symptoms can still transmit the disease to others and can still develop complications later in life. Some people carry the virus in their bodies and are contagious for the rest of their lives [6].

The risk of transmission from mother to newborn can be reduced from 20–90% to 5–10% by administering to the newborn hepatitis B vaccine (HBV) and hepatitis B immune globulin (HBIG) within 12 hours of birth, followed by a second dose of hepatitis B vaccine (HBV) at 1-2 months and a third dose at and not earlier than 6 months (24 weeks) [7, 8]. The hepatitis B infection does not usually require treatment because most adults clear the infection spontaneously [9]. Early antiviral treatment may be required in less than 1% of people whose infection takes a very aggressive course (fulminant hepatitis) or who are immunocompromised. On the other hand, treatment of chronic infection may be necessary to reduce the risk of cirrhosis and liver cancer. Treatment lasts from six months to a year, depending on medication and genotype [10].

One of the primary reasons for studying hepatitis B virus (HBV) infection is to improve control and finally to put down the infection from the population. Mathematical models can be a useful tool in this approach which helps us to optimize the use of finite sources or simply to goal (the incidence of infection) control measures more impressively. Anderson and May [11] used a simple mathematical model to illustrate the effects of carriers on the transmission of HBV. A hepatitis B mathematical model (Medley et al. [4]) was used to develop a strategy for eliminating HBV in New Zealand [5, 12]. Zhao et al. [13] proposed an age structure model to predict the dynamics of HBV transmission and evaluate the long-term effectiveness of the vaccination programme in China. Wang et al. [14] proposed and analyzed the hepatitis B virus infection in a diffusion model confined to a finite domain. Xu and Ma [15] proposed a hepatitis B virus (HBV) model with spatial diffusion and saturation response of the infection rate was investigated. Zou et al. [16] also proposed a mathematical model to understand the transmission dynamics and prevalence of HBV in mainland China. Pang et al. [17] developed a model to explore the impact of vaccination and other controlling measures of HBV infection. Zhang and Zhou [18] proposed analysis and application of an HBV model. Bhattacharyya and Ghosh [19], Kar and Batabyal [20], and Kar and Jana [21] proposed optimal control of infectious diseases.

In this paper, we study the dynamics of hepatitis B virus (HBV) infection under administration of vaccination and treatment, where HBV infection is transmitted in two ways through vertical transmission and horizontal transmission. While the horizontal transmission is reduced through the administration of vaccination to those susceptible individuals, the vertical transmission gets reduced through the administration of treatment to infected individuals; therefore, the vaccine and the treatment play different roles in controlling the HBV [19]. In this study we analyze and apply optimal control to determine the possible impacts of vaccination to susceptible individuals and treatment to infected individuals. Some numerical simulations of the model are also given to illustrate the results and to find optimal strategies in controlling HBV infection.

The paper is organized as follows. In Section 2, we proposed an HBV infection model with vaccination and treatment. In Section 3, we analyzed the qualitative property of the model. In Section 4, we considered the optimal analysis of the model and in Section 5, we considered some numerical experiments under special choice of parameter values. The paper will be finished with a brief discussion and conclusion.

## 2. Model and Preliminaries

To analyze and control hepatitis B virus (HBV) infection in the present paper, we consider a model with two controls: vaccination and treatment. The model is constructed based on the characteristics of HBV transmission and the model of Medley et al. [4]. According to the natural history of HBV, this study has a unique characteristic that distinguishes it from Medley's model as follows. Firstly, in this study, two controlling variables are considered (vaccination and treatment) in order to prevent the spread of the HBV and finally to put down the infection from the population, whereas in Medley's model only one controlling variable, the vaccination, was employed. Secondly, in our model we have considered two different ways of the transmission of hepatitis B virus infection, namely, vertical transmission (hepatitis B virus infection transmits directly from the parents to the offspring) and horizontal transmission (hepatitis B virus infection transmits through contact with infective individuals) [19]. Thirdly, we have considered a proportion of the vaccination in susceptible individuals to all age groups, whereas Medley et al. [4] have utilized only the newborns vaccination. In fact, the adults, especially the teenagers, are encouraged to hepatitis B vaccine [17]. Fourthly, “immunity" has been considered as “permanent" in Medley's model, whereas the effect of the vaccination may be decreased after sometime, and the person may be affected and be susceptible [22]. Consequently we have considered a parameter *λ*_4_ to account for the turning back recovered people to susceptible ones [16, 17].

**Figure 1:**
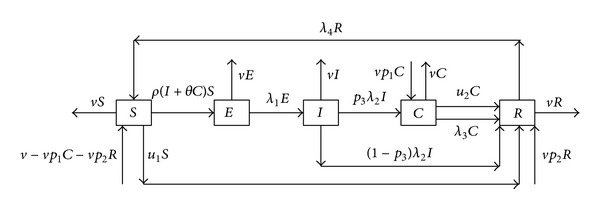
Flow diagram of HBV dynamics under application of vaccine and treatment. *S*, *E*, *I*, *C*, and *R* denote five compartment of susceptible, exposed, acute infection, chronic carriers, and immune or recovered class, respectively. The *ρ*(*I* + *θC*)*S* indicates the horizontal transmission from compartment *S* to *E*, whereas *νp*_1_ *C* denotes the vertical transmission from *C* to *C* by birth of offspring from a carrier individual. Similarly, *νp*_2_ *R* represents the proportion of immune newborn from recovered class. *λ*_3_ *C* shows individuals' spontaneous recovery and move from compartment *C* to *R* and *u*_2_ *C* denotes proportions of carriers move to recovered class by treatment. *λ*_4_ *R* denotes a portion that moves from compartment *R* to *S* due to loss of immunity. *p*_3_ *λ*_2_ *I* shows proportion of acute infection individuals who become carries and (1 − *p*_3_)*λ*_2_ *I* shows proportion of acute infection individuals, clear HBV, and move from *I* to *R*. *u*_1_ *S* denotes proportions of susceptible move to recovered class by vaccination and each compartment has its own death rate.

We consider an *S*-*E*-*I*-*C*-*R* epidemic model by dividing total population into five time-dependent classes, namely, the susceptible individuals *S*(*t*); infected but not yet infectious individuals (exposed) *E*(*t*); acute infection individuals *I*(*t*); chronic HBV carriers *C*(*t*); and recovered *R*(*t*) for hepatitis B virus (HBV) infection that propagates through contact between the infected and the susceptible individuals and also through the infected parents to the offspring. A flow chart of this compartmental model is shown in Figure 1. These assumptions, however, lead to the following dynamic model:

(1) S˙(t)=ν−νp1C−νp2R−ρ(I+θC)S−νS−u1S+λ4R,E˙(t)=ρ(I+θC)S−(ν+λ1)E,I˙(t)=λ1E−(ν+λ2)I,C˙(t)=νp1C+p3λ2I−(ν+λ3)C−u2C,R˙(t)=νp2R+(1−p3)λ2I+λ3C−νR−λ4R+u1S+u2C.

In these equations, all the parameters are nonnegative. We assume stable population with equal per capita birth and death rate *ν* (as disease-induced death rate is not considered in system) and *λ*_1_ is the rate of exposed individuals becoming infections, *λ*_2_ is the rate at which individuals leave the acute infection class, *λ*_3_ is the rate of carrier individuals who recover from the disease by natural way (spontaneous recovery) and move from carrier to recovered [16, 21], and *θ* is the infectiousness of carriers relative to acute infections. A proportion *p*_3_ of acute infection individuals become carriers and another clear HBV moves directly to immunity class [17]. The horizontal transmission of disease propagation is denoted by the mass action term *ρ*(*I* + *θC*)*S*, where *ρ* represents the contact rate. For vertical transmission, we assume that a fraction *p*_1_ of newborns from infected class are infected and it is denoted by the term *νp*_1_ *C*, (*p*_1_ < 1). Similarly, a fraction *p*_2_ of newborns from recovered class are immune and it is denoted by *νp*_2_ *R*, (*p*_2_ < 1) [23]. Consequently, the birth flux into the susceptible class is given by *ν* − *νp*_1_ *C* − *νp*_2_ *R*.

For simplicity, we normalize the population size to 1; that is, now *S*, *E*, *I*, *C*, and *R* are, respectively, the fractions of the susceptible, the exposed, the acute infective, the carriers, and the recovered individuals in the population and *S* + *E* + *I* + *C* + *R* = 1 holds [17]. Hence, the fifth equation may be omitted, and (1) becomes

(2) S˙(t)=ν−νp1C−ρ(I+θC)S−νS−u1S+(λ4−νp2)(1−S−E−I−C),E˙(t)=ρ(I+θC)S−(ν+λ1)E,I˙(t)=λ1E−(ν+λ2)I,C˙(t)=νp1C+p3λ2I−(ν+λ3)C−u2C.

Among them *u*_1_ is the proportion of the susceptible that is vaccinated per unit time; further we assume that the vaccination is not perfect; that is, although vaccination offers a very powerful method of disease control, there are many associated difficulties. Generally, vaccines are not 100% effective, and therefore only a proportion of vaccinated individuals are protected, then some proportion of the vaccinated individuals may be susceptible again to that disease [22]. Similarly, *u*_2_ is the proportion of the chronic HBV carriers that is treated per unit time. The transfer rate from the recovered class to the susceptible class is taken as *λ*_4_ (*λ*_4_ ≥ 0). We assume that the population of newborn carriers born to carriers is less than the sum of the death carriers and the population moving from carrier to recovery state [16]. In this case we have *νp*_1_ < *ν* + *u*_2_ + *λ*_3_; otherwise, carriers would keep increasing rapidly as long as there is infection; that is, *dC*/*dt* > 0 for *C* ≠ 0 or *I* ≠ 0 and *t* ≥ 0.

Let *X*(*t*) = *S*(*t*) + *C*(*t*) + *I*(*t*) + *C*(*t*); then we have

(3) $$\begin{array}{c} \frac{\text{d}X}{dt}=\frac{dS}{dt}+\frac{dE}{dt}+\frac{dI}{dt}+\frac{dC}{dt}; \end{array}$$

that  is,

(4) $$\begin{array}{c} \frac{dX}{dt}+\left(\nu +\lambda_{4}-\nu p_{2}\right)X\leq \nu +\lambda_{4}. \end{array}$$

Now integrating both sides of the above inequality and using the theory of differential inequality [21, 24], we get

(5) $$\begin{array}{c} X\left(t\right)\leq e^{-(\nu +\lambda_{4}-\nu p_{2})t}\left[\frac{\nu +\lambda_{4}}{\nu +\lambda_{4}-\nu p_{2}}e^{(\nu +\lambda_{4}-\nu p_{2})t}+k\right], \end{array}$$

where *k* is a constant and letting *t* → *∞*, we have

(6) S+E+I+C≤ν+λ4ν+λ4−νp2,S˙(t)=ν−νp1C−ρ(I+θC)S−νS−u1S+(λ4−νp2)(1−S−E−I−C);

then

(7) $$\begin{array}{c} S\leq \frac{\nu +\lambda_{4}}{\nu +\lambda_{4}+u_{1}-\nu p_{2}}; \end{array}$$

then

(8) Π={(S,E,I,C)∈ℝ+4 ∣ S≤ν+λ4ν+u1+λ4−νp2, S+E+I+C≤ν+λ4ν+λ4−νp2}

is positively invariant. Hence, the system is mathematically well-posed. There, for initial starting point *x* ∈ ℝ_+_^4^, the trajectory lies in Π. Therefore, we will focus our attention only on the region Π.

## 3. Analysis of the System for Constant Controls

In this section, we assume that the control parameters *u*_1_(*t*) and *u*_2_(*t*) are constant functions.

### 3.1. Equilibrium Points and Basic Reproduction Number

We will discuss the existence of all the possible equilibrium points of the system (2). We see that system (2) has two possible nonnegative equilibria.

(i) Disease-free equilibrium point *E*_0_ = (*S*_0_, 0,0, 0), where

(9) $$\begin{array}{c} S_{0}=\frac{{\nu -\nu p_{2}+\lambda }_{4}}{u_{1}+\nu +\lambda_{4}-\nu p_{2}}; \end{array}$$

it is always feasible. In the absence of vaccination, this is reduced to the equilibrium (1,0, 0,0).

**Definition**

The basic reproduction number, denoted by *R*_0_, is the expected number of secondary cases produced, in a completely susceptible population, by a typical infective individual [25].

Using the notation in van den Driessche and Watmough [25], we have

(10) F=[0ρS0ρθS0000000]V=[ν+λ100−λ1ν+λ200−p3λ2ν+λ3+u2−νp1].

The reproduction number is given by *ρ*(*FV* ^−1^), and

(11) R0=ρλ1(ν+λ3+u2−p1ν+θp3λ2)(ν−νp2+λ4)(ν+λ1)(ν+λ2)(ν+λ3+u2−p1ν)(u1+ν+λ4−νp2)=ρλ1(ν+λ3+u2−p1ν+θp3λ2)(ν+λ1)(ν+λ2)(ν+λ3+u2−p1ν)S0=ρλ1(ν+λ1)(ν+λ2)[1+θp3λ2(ν+λ3+u2−νp1)]S0=ρλ1(ν+λ1)(ν+λ2) ×[1+θp3λ2(ν+λ3+u2−νp1)][ν−νp2+λ4u1+ν+λ4−νp2].

Define

(12) R1=ρλ1S0(ν+λ1)(ν+λ2),R2=ρθp3λ1λ2S0(ν+λ1)(ν+λ2)(ν+λ3+u2−νp1);

then we can see that *R*_0_ = *R*_1_ + *R*_2_.

**Remark**

**Figure 2:**
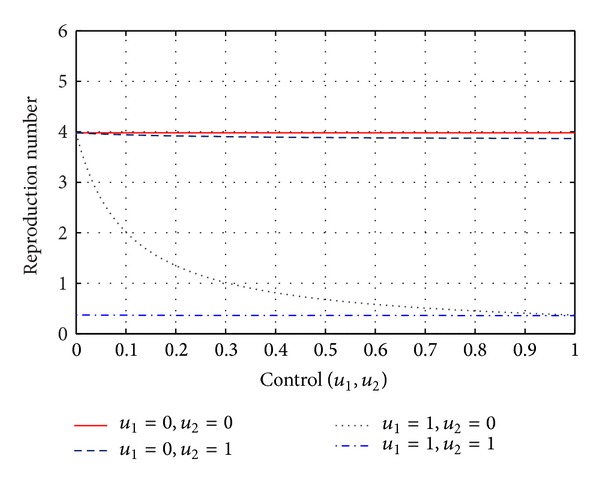
Variation of different reproduction numbers by using of two control variables (vaccination and treatment). Figure 2 illustrates the impact of application of two controls (vaccination and treatment) on different basic reproduction numbers. *R*_0_ = 3.9810, when there is no vaccination and treatment (*u*_1_ = 0; *u*_2_ = 0). The figure shows that application of vaccination reduces *R*_0_ more rapidly than treatment, though mixed intervention strategies always work better to reduce the disease burden.

We should note from (11) that the use both of vaccine and treatment control to both reduce the value of *R*_0_, and at the same time effects of both intervention strategies on *R*_0_ are not simply the addition of two independent effects, rather they multiply together in order to improve the overall effects of population level independently (Figure 2). Consider the following:

(ii) endemic equilibria, E** = (S*, E*, I*, C*), which has four different cases.

(a) No vaccine, no treatment control (*u*_1_ = 0, *u*_2_ = 0), *E*_1_** = (*S*_1_*, *E*_1_*, *I*_1_*, *C*_1_*), where
  (13) S1∗= (ν+λ1)(ν+λ2)(ν+λ3−p1ν)ρλ1(ν+λ3+θλ2p3−p1ν),E1∗= θρ(ν+λ2)C1∗S1∗(ν+λ1)(ν+λ2)−ρλ1S1∗,I1∗= θρλ1C1∗S1∗(ν+λ1)(ν+λ2)−ρλ1S1∗,C1∗=(λ1λ2p3(ν+λ4−νp2)S1∗(R0−1))×((ν+λ3−p1ν)  ×[(ν+λ4−νp2)(ν+λ2+λ1)+λ1λ2]  +λ1λ2p3(νp1−νp2+λ4))−1.
(b) No vaccine, with treatment control (*u*_1_ = 0, *u*_2_ ≠ 0), *E*_2_** = (*S*_2_*, *E*_2_*, *I*_2_*, *C*_2_*), where
  (14) S2∗= (ν+λ1)(ν+λ2)(ν+λ3−p1ν+u2)ρλ1(ν+λ3+θλ2p3−p1ν+u2),E2∗= θρ(ν+λ2)C2∗S2∗(ν+λ1)(ν+λ2)−ρλ1S2∗,I2∗= θρλ1C2∗S2∗(ν+λ1)(ν+λ2)−ρλ1S2∗,C2∗=(λ1λ2p3(ν+λ4−νp2)S2∗(R0−1))×((ν+λ3−p1ν+u2)  ×[(ν+λ4−νp2)(ν+λ2+λ1)+λ1λ2]  +λ1λ2p3(νp1−νp2+λ4))−1.
(c) With vaccine, no treatment control (*u*_1_ ≠ 0, *u*_2_ = 0), *E*_3_** = (*S*_3_*, *E*_3_*, *I*_3_*, *C*_3_*), where
  (15) S3∗= (ν+λ1)(ν+λ2)(ν+λ3−p1ν)ρλ1(ν+λ3+θλ2p3−p1ν),E3∗= θρ(ν+λ2)C3∗S3∗(ν+λ1)(ν+λ2)−ρλ1S3∗,I3∗= θρλ1C3∗S3∗(ν+λ1)(ν+λ2)−ρλ1S3∗,C3∗=(λ1λ2p3(ν+λ4−νp2+u1)S3∗(R0−1))×((ν+λ3−p1ν)  ×[(ν+λ4−νp2)(ν+λ2+λ1)+λ1λ2]  +λ1λ2p3(νp1−νp2+λ4))−1.
(d) With vaccine, with treatment control (*u*_1_ ≠ 0, *u*_2_ ≠ 0), *E*_4_** = (*S*_4_*, *E*_4_*, *I*_4_*, *C*_4_*), where
  (16) S4∗= (ν+λ1)(ν+λ2)(ν+λ3−p1ν+u2)ρλ1(ν+λ3+θλ2p3−p1ν+u2),E4∗= θβ(ν+λ2)C4∗S4∗(ν+λ1)(ν+λ2)−ρλ1S4∗,I4∗= θρλ1C4∗S4∗(ν+λ1)(ν+λ2)−ρλ1S4∗,C4∗=(λ1λ2p3(ν+λ4−νp2+u1)S4∗(R0−1))×((ν+λ3−p1ν+u2)  ×[(ν+λ4−νp2)(ν+λ2+λ1)+λ1λ2]  +λ1λ2p3(νp1−νp2+λ4))−1.

Clearly E** is feasible if C* > 0, that is, if *R*_0_ > 1. Also for *R*_0_ = 1, the endemic equilibrium reduces to the disease-free equilibrium and for *R*_0_ < 1 it becomes infeasible. Hence we may state the following theorem.

**Theorem**

(i) If  *R*_0_ < 1, then the system (2) has only one equilibrium, which is disease-free.

(ii) If  *R*_0_ > 1, then the system (2) has two equilibria: one is disease-free and the other is endemic equilibrium.

(iii) If  *R*_0_ = 1, then the endemic equilibrium reduces to the disease-free equilibrium.

### 3.2. Stability Analysis

In this section, we will discuss the stability of different equilibria. Firstly, we analyse the local stability of the disease-free equilibrium.

**Theorem**

If *R*_0_ < 1, then the disease-free equilibrium is locally asymptotically stable.

**Proof**

The Jacobian matrix of system (2) at the disease-free equilibrium is

(17) $$\begin{array}{c} J_{0}=\left[\begin{array}{cccc} -\left(\nu +\lambda_{4}-\nu p_{2}+u_{1}\right) & -\left(\lambda_{4}-\nu p_{2}\right) & -\left(\rho S_{0}+\lambda_{4}-\nu p_{2}\right) & -\left(\nu p_{1}+\lambda_{4}-\nu p_{2}+\theta \rho S_{0}\right)\\ 0 & -\left(\nu +\lambda_{1}\right) & \rho S_{0} & \theta \rho S_{0}\\ 0 & \lambda_{1} & -\left(\nu +\lambda_{2}\right) & 0\\ 0 & 0 & p_{3}\lambda_{2} & \nu p_{1}-\nu -\lambda_{3}-u_{2} \end{array}\right]. \end{array}$$

The characteristic polynomial of *J*_0_ given by

(18) $$\begin{array}{c} P\left(\lambda \right)=\left(\lambda +l_{0}\right)\left(\lambda^{3}+l_{1}\lambda^{2}+l_{2}\lambda +l_{3}\right), \end{array}$$

where

(19) l0= ν+u1+λ4−νp2,l1= 3ν+λ1+λ2+λ3+u2−νp1,l2=(ν+λ2)(ν+λ3+u2−νp1)+(ν+λ1)(2ν+λ2+λ3+u2−νp1)−λ1ρS0,l3=(ν+λ1)(ν+λ2)(ν+λ3+u2−νp1)−λ1ρS0(ν+λ3+u2+θp3λ2−νp1).

We need to verify the following two conditions:

- *l*_0_, *l*_1_, *l*_2_, *l*_3_ > 0;
- *l*_1_ *l*_2_ − *l*_3_ > 0.

It is easy to see that *l*_0_, *l*_1_ > 0 and *l*_2_, *l*_3_, *l*_1_ *l*_2_ − *l*_3_ > 0 if *R*_0_ < 1. It follows from the Routh-Hurwitz criterion that the eigenvalues have negative real parts if *R*_0_ < 1. Hence, the disease-free equilibrium of model (2) is locally asymptotically stable if *R*_0_ < 1 and unstable if *R*_0_ > 1.

To discuss the properties of the endemic equilibrium point

**Theorem**

The endemic equilibrium point is locally asymptotically stable if *R*_0_ > 1.

**Proof**

We use Routh-Hurwitz criterion to establish the local stability of the endemic equilibrium. The Jacobian matrix of system (2) at endemic equilibrium is

(20) $$\begin{array}{c} J^{*}=\left[\begin{array}{cccc} -\left(\nu +\lambda_{4}-\nu p_{2}+u_{1}+\rho I^{*}\right) & -\left(\lambda_{4}-\nu p_{2}\right) & -\left(\rho S^{*}+\lambda_{4}-\nu p_{2}\right) & -\left(\nu p_{1}+\lambda_{4}-\nu p_{2}+\rho \theta S^{*}\right)\\ \rho I^{*}+\rho \theta C^{*} & -\left(\nu +\lambda_{1}\right) & \rho S^{*} & \rho \theta S^{*}\\ 0 & \lambda_{1} & -\left(\nu +\lambda_{2}\right) & 0\\ 0 & 0 & p_{3}\lambda_{2} & \nu p_{1}-\nu -\lambda_{3}-u_{2} \end{array}\right]. \end{array}$$

Then the characteristic equation at E** is *λ* ^4^ + *f*_1_ *λ* ^3^ + *f*_2_ *λ* ^2^ + *f*_3_ *λ* + *f*_4_ = 0, where

(21) f1= a1+b2+c3+d4,f2= a1a3+a1d4+a1b2+c3d4+b2c3+b2d4−b3c2−b1a2,f3=a1c3d4+a1b2c3+a1b2d4+b2c3d4+b4c2d3+b1c2a3−a1b3c2−b3c2d4−b1a2c3−b1a2d4,f4=a1b2c3d4+a1b4c2d3+b1c2a3d4−a1b3c2d4−b1a2c3d4−b1a4c2d3,

where

(22) a1 =−(ν+λ4−νp2+u1+ρI∗),a2 =−(λ4−νp2),  a3=−(ρS∗+λ4−νp2),a4 =−(νp1+λ4−νp2+ρθS∗),  b1=ρI∗+ρθC∗,b2 =−(ν+λ1),  b3=ρS∗,b4 =ρθS∗,  c1=0,c2 =λ1,  c3=−(ν+λ2),c4 =0,  d1=0,d2 =0,  d3=p3λ2,d4 =νp1−ν−λ3−u2.

We need to verify the following three conditions:

- *f*_1_, *f*_2_, *f*_3_, *f*_4_ > 0;
- *f*_1_ *f*_2_ − *f*_3_ > 0;
- *f*_3_(*f*_1_ *f*_2_ − *f*_3_) − *f*_1_^2^ *f*_4_ > 0.

It is easy to see that conditions (a) and (b) are satisfied. After computations, we can prove that *f*_3_(*f*_1_ *f*_2_ − *f*_3_) − *f*_1_^2^ *f*_4_ > 0 is also valid. The Routh-Hurwitz criterion and those inequalities in (a)–(c) imply that the characteristic equation at E** has only roots with negative real part, which certifies the local stability of E**.

## 4. Optimal Control with Two Objectives

One of the early reasons for studying hepatitis B virus (HBV) infection is to improve the control variables and finally to put down the infection of the population. Optimal control is a useful mathematical tool that can be used to make decisions in this case. In the previous sections we have analyzed the model with two control variables, one is treatment and the other is vaccination and we consider their constant controls throughout the analysis. But in fact these control variables should be time dependent. In this section we consider the vaccination and treatment as time-dependent controls in a compact interval of time duration.

Our goals here are to put down infection from the population by increasing the recovered individuals and reducing susceptible, exposed, infected, and carrier individuals in a population and to minimize the costs required to control the hepatitis B virus (HBV) infection by using vaccination and treatment. First of all, we construct the objective functional to be optimized as follows:

(23) J=∫0T[A1S(t)+A2E(t)+A3I(t)+A4C(t)   +12(B1u12(t)+B2u22(t))]dt

subject to

(24) S˙(t)=ν−νp1C−ρ(I+θC)S−νS−u1S+(λ4−νp2)(1−S−E−I−C),E˙(t)= ρ(I+θC)S−(ν+λ1)E,I˙(t)= λ1E−(ν+λ2)I,C˙(t)= νp1C+p3λ2I−(ν+λ3)C−u2C.

Our object is to find (*u*_1_*, *u*_2_*) such that

(25) $$\begin{array}{c} J\left(u_{1}^{*},u_{2}^{*}\right)=\min J\left(u_{1},u_{2}\right), \end{array}$$

where

(26) u1(t),  u2(t)∈Γ ={u(t) ∣ 0≤u(t)≤umax⁡(t)≤1,  0≤t≤T,   u(t)  is  Labesgue  measurable}.

Here *A*_*i*_, for *i* = 1,…, 4 are positive constants that are represented to keep a balance in the size of *S*(*t*), *E*(*t*), *I*(*t*), and *C*(*t*), respectively; *B*_1_ and *B*_2_, respectively, are the weights corresponding to the controls *u*_1_ and *u*_2_. *u*_max⁡_ is the maximum attainable value for controls (*u*_1max⁡_ and *u*_2max⁡_); *u*_1max⁡_ and *u*_2max⁡_ will depend on the amount of resources available to implement each of the control measures [26]. The *B*_1_ and *B*_2_ will depend on the relative importance of each of the control measures in mitigating the spread of the disease as well as the cost (human effort, material resources, etc.) of implementing each of the control measures per unit time [26]. Thus, the terms *B*_1_ *u*_1_^2^ and *B*_2_ *u*_2_^2^ describe the costs associated with vaccination and treatment, respectively. The square of the control variables is taken here to remove the severity of the side effects and overdoses of vaccination and treatment [27].

### 4.1. Optimal Control Solution

For existence of the solution, we consider the control system (24) with initial condition

(27) $$\begin{array}{c} S\left(0\right)=S_{0},\;\;E\left(0\right)=E_{0},\;\\ I\left(0\right)=I_{0},\;\;C\left(0\right)=C_{0}; \end{array}$$

then, we rewrite (2) in the following form:

(28) $$\begin{array}{c} \frac{dx\left(t\right)}{dt}=Ax+F\left(x\right), \end{array}$$

where *x*(*t*) = (*S*(*t*), *E*(*t*), *I*(*t*), *C*(*t*)) is the vector of the state variables and **A** and **F**(**x**) are defined as follows:

(29) A=[−ν−u1−λ4+νp2−λ4+νp2−λ4+νp2−λ4+νp20−ν−λ1000λ1−ν−λ2000p3λ2νp1−ν−λ3−u2],F(x)=[ν+ρ(I+θC)S+λ4−νp2ρ(I+θC)S00].

We set

(30) $$\begin{array}{c} G\left(x\right)=Ax+F\left(x\right). \end{array}$$

The second term on the right-hand side of (30) satisfies

(31) |F(x1)−F(x2)| ≤M(|S1−S2|+|E1−E2|+|I1−I2|+|C1−C2|),

where the positive constant *M* is independent of the state variables *x* and *M* ≤ 1. Also, we get

(32) $$\begin{array}{c} \left|G\left(x_{1}\right)-G\left(x_{2}\right)\right|\leq L\left|x_{1}-x_{2}\right|, \end{array}$$

where *L* = max⁡{*M*, ||*A*||} < *∞*. Therefore, it follows that the function *G* is uniformly Lipschitz continuous. From the definition of the controls *u*_1_(*t*) and *u*_2_(*t*) and the restrictions on the nonnegativeness of the state variables we see that a solution of the system (28) exists [24, 28, 29].

### 4.2. The Lagrangian and Hamiltonian for the Control Problem

In order to find an optimal solution pair, first we should find the Lagrangian and Hamiltonian for the optimal control problem (24). In fact, the Lagrangian of problem is given by

(33) L(S,E,I,C,u1,u2)=A1S(t)+A2E(t)+A3I(t)+A4C(t)+12(B1u12(t)+B2u22(t)).

We are looking for the minimal value of the Lagrangian. To accomplish this, we define Hamiltonian *H* for the control problem as follows:

(34) H(S,E,I,C,u1,u2,α1,α2,α3,α4)  =L+α1(t)dSdt+α2(t)dEdt+α3(t)dIdt+α4(t)dCdt,

where *α*_*i*_(*t*) for *i* = 1,2, 3,4 are the adjoint variables and can be determined by solving the following system of differential equations:

(35) α˙1(t)=−∂H∂S=−[A1+α1((νp2−λ4)−ρ(I+θC)−u1−ν)  +α2ρ(I+θC)],α˙2(t)=−∂H∂E=−[A2+α1(νp2−λ4)+α2(−ν−λ1)+α3λ1],α˙3(t)=−∂H∂I=−[A3+α1((νp2−λ4)−ρS)+α2ρS   +α3(−ν−λ2)+α4p3λ2],α˙4(t)=−∂H∂C=−[A4+α1(νp2−λ4−νp1−ρθS)   +α2ρθS+α4(νp1−ν+λ3+u2)],

satisfying the transversality conditions and optimality conditions:

(36) α1(T)=α2(T)=α3(T)=α4(T)=0,∂H∂u1=B1u1−α1S=0,∂H∂u2=B2u2−α4C=0.

We now state and prove the following theorem.

**Theorem**

There is an optimal control (*u*_1_*, *u*_2_*) such that

(37) $$\begin{array}{c} J\left(S,E,I,S,u_{1}^{*},u_{2}^{*}\right)=\min J\left(S,E,I,S,u_{1},u_{2}\right) \end{array}$$

subject to the system of differential equation (24).

**Proof**

Here both the control and state variables are nonnegative values. In this minimized problem, the necessary convexity of the objective functional in *u*_1_ and *u*_2_ are satisfied. The set of all control variables *u*_1_(*t*), *u*_2_(*t*) is also convex and closed by definition. The optimal system is bounded which determines the compactness needed for the existence of the optimal control. In addition, the integrand in the functional

(38) ∫0T[A1S(t)+A2E(t)+A3I(t)+A4C(t)  +12(B1u12(t)+B2u22(t))]dt

is convex on the control set. Hence the theorem (the proof is complete).

### 4.3. Necessary and Sufficient Conditions for Optimal Controls

Applying Pontryagin's maximum principle [30] on the constructed Hamiltonian *H*, and the theorem (24), we obtain the optimal steady-state solution and corresponding control as follows: If taking *X* = (*S*, *E*, *I*, *C*) and *U* = (*u*_1_, *u*_2_), then (X*, U*) is an optimal solution of an optimal control problem; then we now state the following theorem.

**Theorem**

The optimal control pair (*u*_1_*, *u*_2_*) which minimizes J over the region Γ is given by

(39) $$\begin{array}{c} u_{1}^{*}=\underset{\left[0,T\right]}{\max}\left\{0,\min\left({\tilde{u}}_{1}\left(t\right),1\right)\right\},\\ u_{2}^{*}=\underset{\left[0,T\right]}{\max}\left\{0,\min\left({\tilde{u}}_{2}\left(t\right),1\right)\right\}, \end{array}$$

where ${\tilde{u}}_{1}={\tilde{\alpha }}_{1}S/B_{1}$ and ${\tilde{u}}_{2}={\tilde{\alpha }}_{4}C/B_{2}$ and let $\left\{{\tilde{\alpha }}_{1},{\tilde{\alpha }}_{2},{\tilde{\alpha }}_{3},{\tilde{\alpha }}_{4}\right\}$ be the solution of system (35).

**Proof**

With the help of the optimality conditions, we have

(40) $$\begin{array}{c} \frac{\partial H}{\partial u_{1}}=B_{1}u_{1}-\alpha_{1}S=0⟹u_{1}=\frac{{\tilde{\alpha }}_{1}S}{B_{1}}=\left({\tilde{u}}_{1}\right),\\ \frac{\partial H}{\partial u_{2}}=B_{2}u_{2}-\alpha_{4}C=0⟹u_{2}=\frac{{\tilde{\alpha }}_{4}C}{B_{2}}=\left({\tilde{u}}_{2}\right). \end{array}$$

Using the property of the control space Γ, the two controls which are bounded with upper and lower bounds are, respectively, 1 and 0; that is,

(41) $$\begin{array}{c} u_{1}^{*}=\{\begin{array}{cc} 0 & \text{if}\;\;{\tilde{u}}_{1}\leq 0\\ {\tilde{u}}_{1} & \text{if}\;\;0<{\tilde{u}}_{1}<1\\ 1 & \text{if}\;\;{\tilde{u}}_{1}\geq 1. \end{array} \end{array}$$

This can be rewritten in compact notation

(42) $$\begin{array}{c} u_{1}^{*}=\underset{\left[0,T\right]}{\max}\left\{0,\min\left({\tilde{u}}_{1}\left(t\right),1\right)\right\}, \end{array}$$

and similarly

(43) $$\begin{array}{c} u_{2}^{*}=\{\begin{array}{cc} 0 & \text{if}\;\;{\tilde{u}}_{2}\leq 0\\ {\tilde{u}}_{2} & \text{if}\;\;0<{\tilde{u}}_{2}<1\\ 1 & \text{if}\;\;{\tilde{u}}_{2}\geq 1. \end{array} \end{array}$$

This can be rewritten in compact notation

(44) $$\begin{array}{c} u_{2}^{*}=\underset{\left[0,T\right]}{\max}\left\{0,\min\left({\tilde{u}}_{2}\left(t\right),1\right)\right\}. \end{array}$$

Hence for these pair of controls (*u*_1_*, *u*_2_*) we get the optimum value of the functional *J* given by (24).

## 5. Numerical Examples

Numerical solutions to the optimality system (24) are discussed in this section. We make several interesting observation by numerically simulating (2) in the range of parameter values. We consider the parameter set Δ = {*ν*, *ρ*, *θ*, *λ*_1_, *λ*_2_, *λ*_3_, *λ*_4_, *p*_1_, *p*_2_, *p*_3_, *A*_1_, *A*_2_, *A*_3_, *A*_4_, *B*_1_, *B*_2_}; some of the parameters are taken from the published articles and some are assumed with feasible values. Moreover, the time interval for which the optimal control is applied is taken as 70 years; also consider Ψ = {*S*_0_, *E*_0_, *I*_0_, *C*_0_} as initial condition for simulation of the model. The main parameter values are listed in Table 1. We compare the results having no controls, only vaccination control, only treatment control, and both vaccination and treatment controls.

With the parameter values in Table 1, the system asymptotically approaches towards the equilibrium *E*_1_**(0.0858,0.3327,0.4003,0.5349), where the basic reproduction ratio *R*_0_ = 3.9810 (Figure 2). For the parameters set the system (2) has two feasible equilibria; one is disease free and the other is endemic, and the endemic equilibrium is locally asymptotically stable. The effect of two control measures on disease dynamics may be understood well if we consider Figure 2. It explains how control reproduction ratio *R*_0_ evolves with different rates of *u*_1_ and *u*_2_. It is seen that both vaccination and treatment reduce the value of *R*_0_ effectively. But an integrated control works better than either of the control measures.

In this section, we use an iterative method to obtain results for an optimal control problem of the proposed model (24). We use Runge-Kutta's fourth-order procedure [23] here to solve the optimality system consisting of eight ordinary differential equations having four state equations and four adjoint equations and boundary conditions. Because state equations have initial conditions and adjoint equations have conditions at the final time, an iterative program was created to numerically simulate solutions. Given an initial guess for the controls, to compute the optimal state values, the program solves (24) with initial conditions (27) forward in time interval [0,70] using a Runge Kutta method of the fourth order. Resulting state values are placed in adjoint equations (35). These adjoint equations with given final conditions are then solved backwards in time. Again, a fourth order Runge Kutta method is employed. Both state and adjoint values are used to update the control using the characterization (39) and the entire process repeats itself. This iterative process terminates when current state, adjoint, and control values are sufficiently close to successive values. Then we use the backward Runge-Kutta fourth-order procedure to solve the adjoint variables in the same time interval with the help of the solution of the state variables transversality conditions.

We have plotted susceptible, exposed, acute infection individuals, and carriers individuals with and without control by considering values of parameters. We simulate the system at different values of rate of *u*_1_ and *u*_2_.

**Figure 3:**
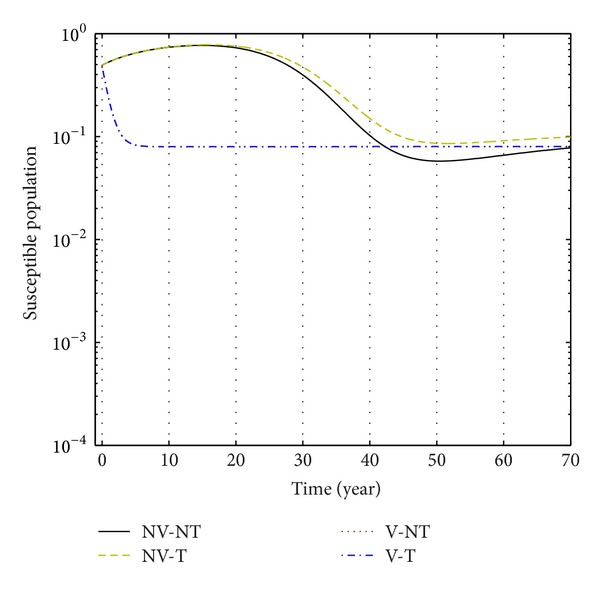
The plot shows the changes in sensitive populations (without) vaccination and (without) treatment.

**Figure 4:**
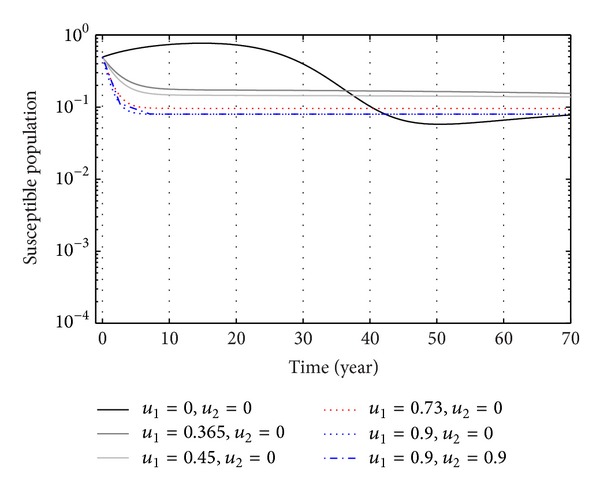
The plot shows the sensitivity of sensitive populations for different values of control *u*_1_.

**Figure 5:**
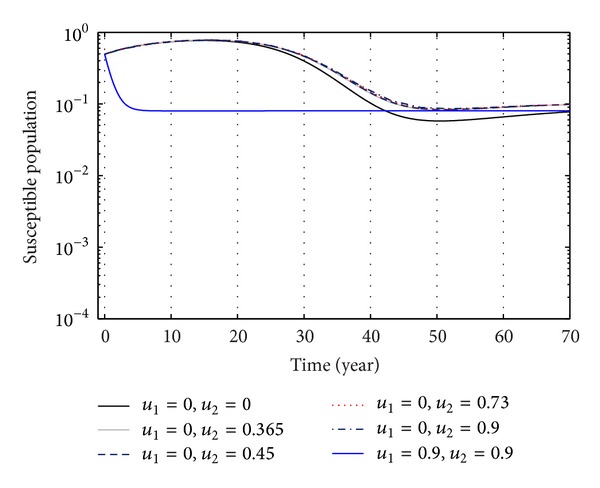
The plot shows the sensitivity of sensitive populations for different values of control *u*_2_.

From Figures 3, 4, and 5, we see that after 20 years the number of susceptible population decreases than when there is no control. In this case most of this population tends to the infected class. Again when only treatment control is applied, then the number of susceptible population is not much different than the population in the case having no control. But the susceptible population differs much from these two strategies if we apply the strategies of only vaccination control and both vaccination and treatment controls. At a high rate of vaccination, the sensitive population density is reduced to a very low level initially and then it takes longer time to restore the steady-state value.

**Figure 6:**
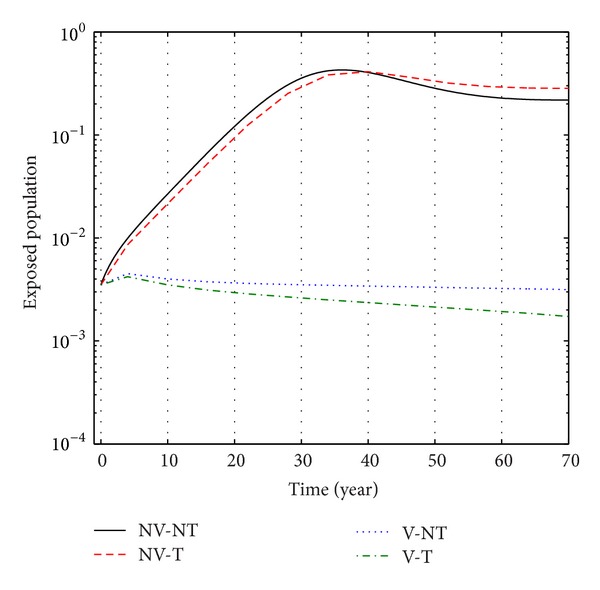
The plot shows the changes in exposed populations (without) vaccination and (without) treatment.

**Figure 7:**
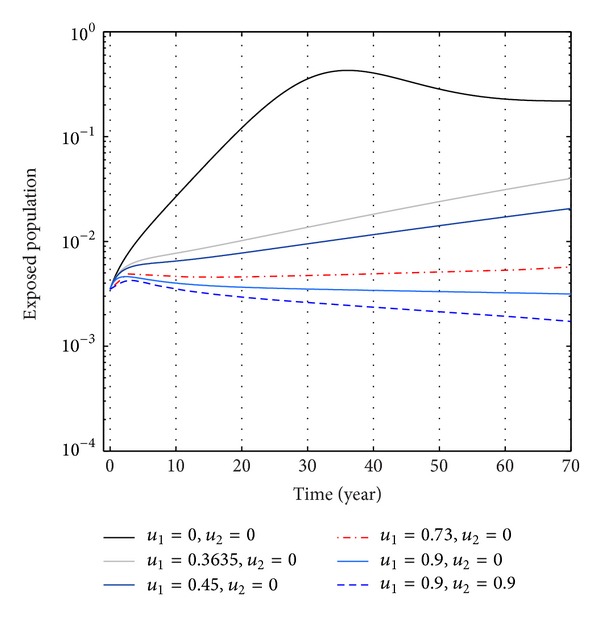
The plot shows the sensitivity of exposed populations for different values of control *u*_1_.

**Figure 8:**
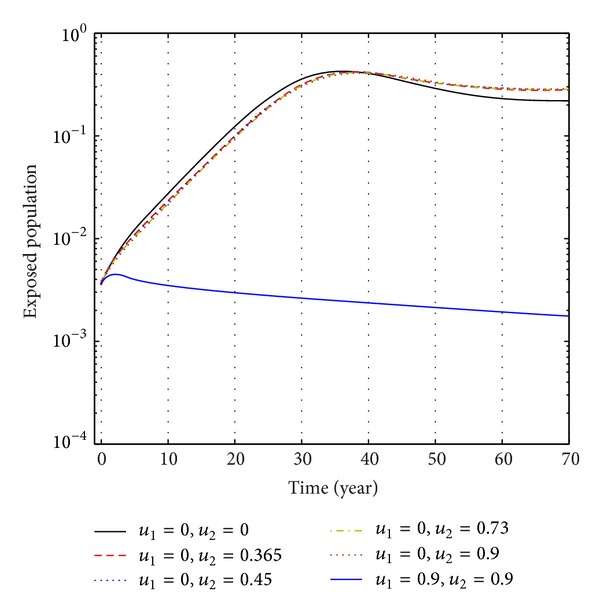
The plot shows the sensitivity of exposed populations for different values of control *u*_2_.

From Figures 6, 7, and 8, we see that the number of exposed population increase than when there is no control. In this case, most of this population tends to the acute infected class. Again when only treatment control is applied, then the number of exposed population is not much different than the population in the case having no control. But the exposed population differs much from these two strategies if we apply the strategies of only vaccination control and both vaccination and treatment controls.

**Figure 9:**
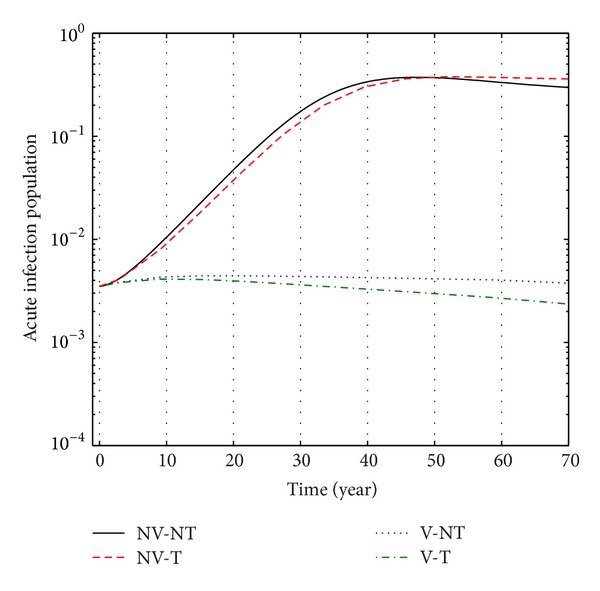
The plot shows the changes in acute infection populations (without) vaccination and (without) treatment.

**Figure 10:**
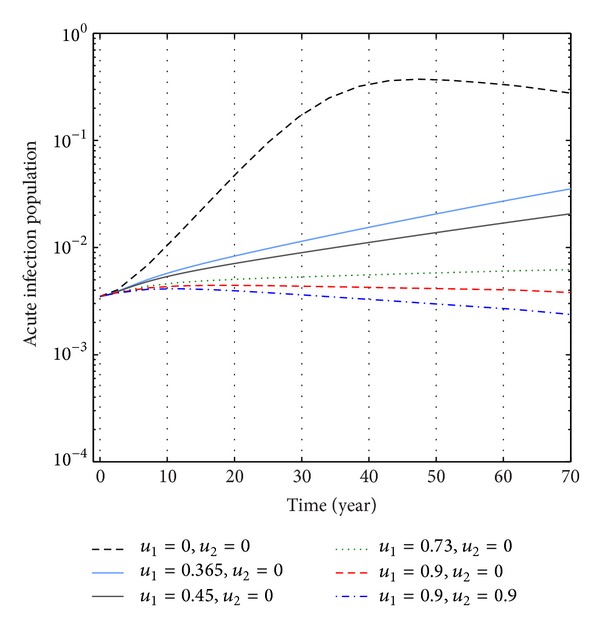
The plot shows the sensitivity of acute infection populations for different values of control *u*_1_.

**Figure 11:**
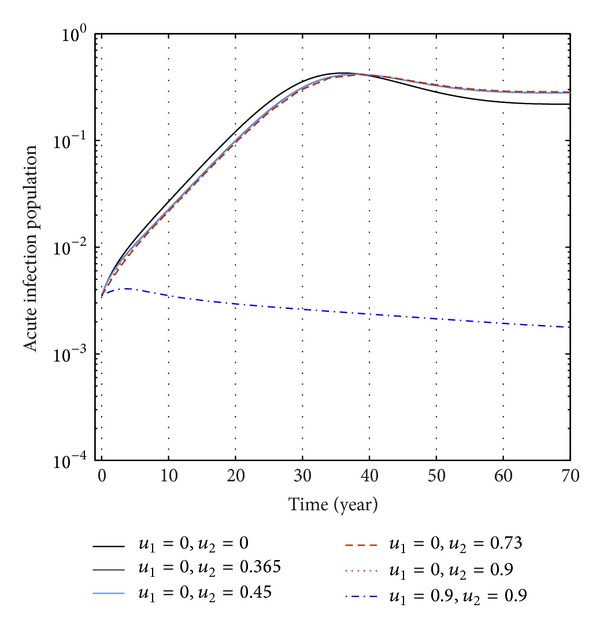
The plot shows the sensitivity of acute infection populations for different values of control *u*_2_.

From Figures 9, 10, and 11, we see that the number of acute infected population increases than when there is no control. In this case most of this population tends to the carrier class. Again when only treatment control is applied, then the number of acute infected population is not much different than the population in the case having no control. But the acute infected population differs much from these two strategies if we apply the strategies of only vaccination control and both vaccination and treatment controls.

**Figure 12:**
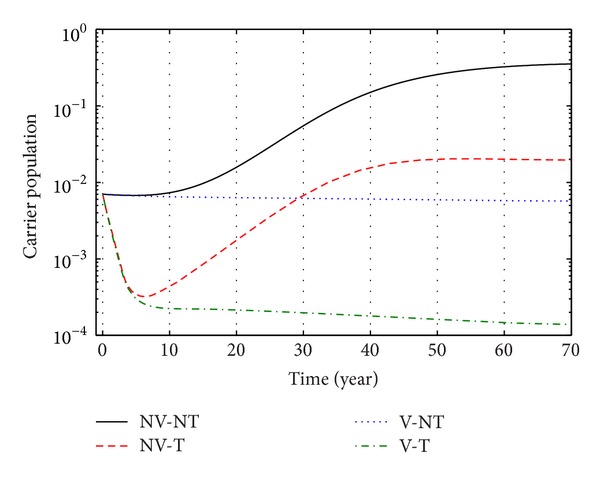
The plot shows the changes in carrier populations (without) vaccination and (without) treatment.

**Figure 13:**
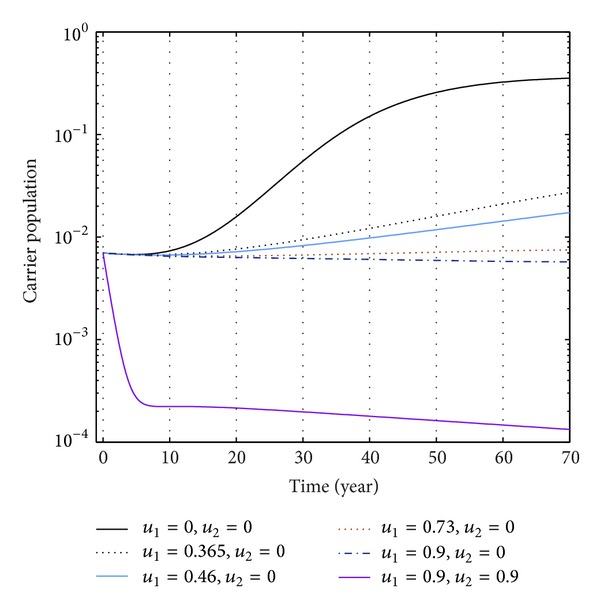
The plot shows the sensitivity of carrier populations for different values of control *u*_1_.

**Figure 14:**
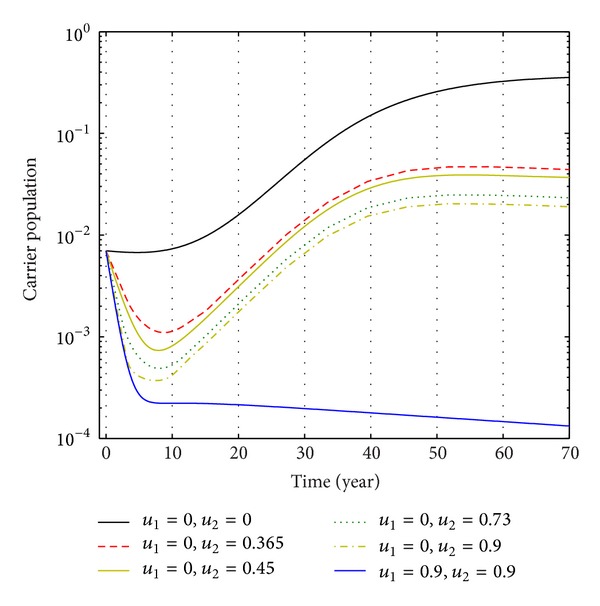
The plot shows the sensitivity of carrier populations for different values of control *u*_2_.

Again from Figures 12, 13, and 14, we see that the number of carrier population increases than when there is no control. We see that the application of only vaccination control gives better result than the application of no control. Again application of treatment control would give better result than the application of vaccination control since the treatment control is better than the vaccination control while the application of both vaccination and treatment controls give the best result as in this case the number of carrier population would be the least in number.

Finally, the effect of two control measures on disease dynamics may be understood well if we consider Figures 1–14, 15, 16, 17, and 18. Since our main purpose is to reduce the number of sensitive, exposed, acute infection, and carrier individuals, therefore numerical simulation results show that optimal combination of vaccination and treatment is the most effective way to control hepatitis B virus infection.

## 6. Conclusion and Suggestions

In this paper, we propose an S-*E*-*I*-*C*-*R* model of hepatitis B virus infection with two controls: vaccination and treatment. First we analyze the dynamic behavior of the system for constant controls. In the constant controls case, we calculate the basic reproduction number and investigate the existence and stability of equilibria. There are two nonnegative equilibria of the system, namely, the disease-free and endemic. We see that the disease-free equilibrium which always exists and is locally asymptotically stable if *R*_0_ < 1, and endemic equilibrium which exists and is locally asymptotically stable if *R*_0_ > 1.

After investigating the dynamic behavior of the system with constant controls we formulate an optimal control problem if the controls become time dependent and solve it by using Pontryagin's maximum principle. Different possible combinations of controls are used and their effectiveness is compared by simulation works. Also, from the numerical results it is very clear that a combination of mixed control measures respond better than any other independent control.

There is still a tremendous amount of work needed to be done in this area. Parameters are rarely constant because they depend on environmental conditions. We do not know, however, the detailed relationship between these parameters and environmental conditions. There may be a time lag as a susceptible population may take some time to be infected and also a susceptible population may take some time to immune after vaccination. We leave all these possible extensions for the future work.

## Figures and Tables

**Figure 15:**
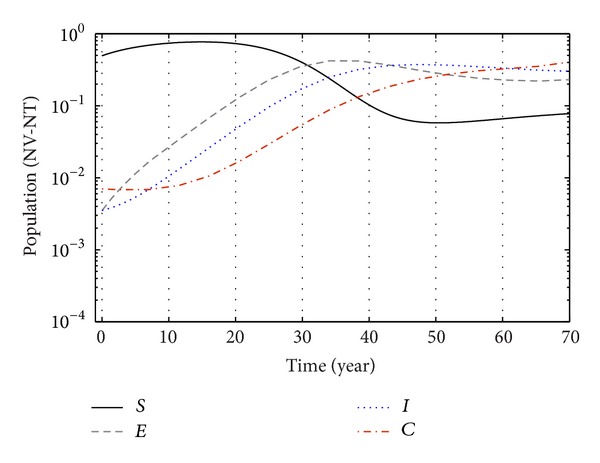
The plot shows the changes in total populations without vaccination and without treatment.

**Figure 16:**
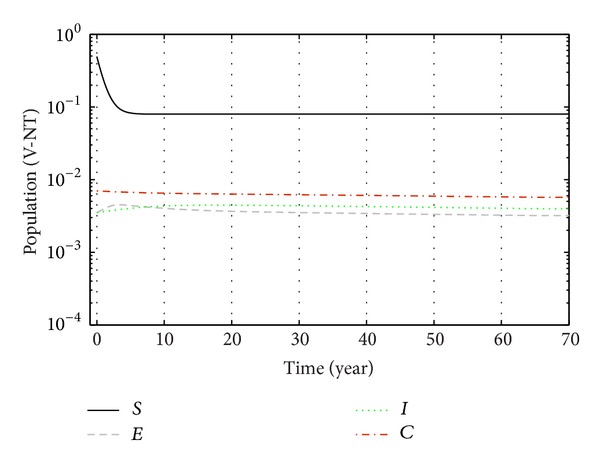
The plot shows the changes in total populations with vaccination and without treatment.

**Figure 17:**
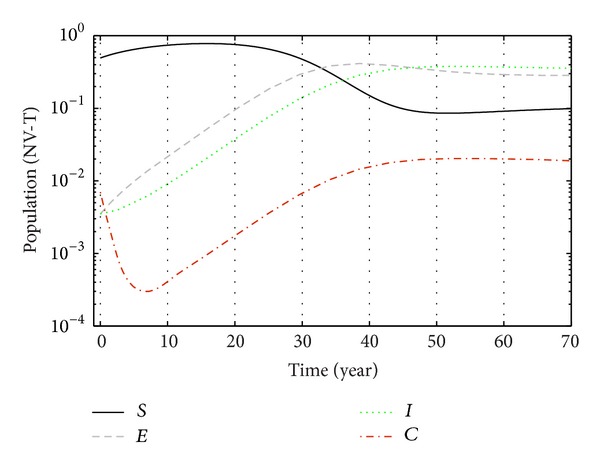
The plot shows the changes in total populations without vaccination and with treatment.

**Figure 18:**
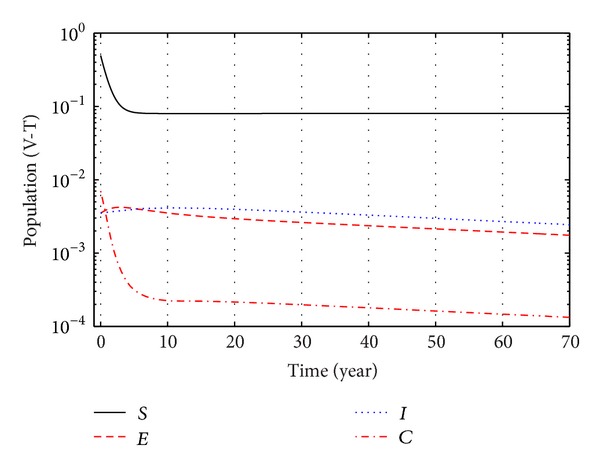
The plot shows the changes in total populations with vaccination and treatment.

**Table 1:** Parameter values used in numerical simulations.

Parameter	Description	Values range	Reference
ν	Birth (and death) rate	0.0121	[17, 18]
*ρ*	Transmission rate	0.8–20.49	[17]
θ	Infectiousness of carriers relative to acute infections	0-1	[17]
λ_1_	Rate of moving from exposed to acute	6 per year	[16]
λ_2_	Rate at which individuals leave the acute infection class	4 per year	[16]
λ_3_	Rate of moving from carrier to recovery	0.025 per year	[16]
λ_4_	Loss of recovery rate	0.03–0.06	[17]
*P*_1_	Probability of infected newborns	0.11	[19]
*P*_2_	Probability of immune newborns	0.1	[19]
*P*_3_	Proportion of acute infection individuals becoming carriers	0.05–0.9	[17]
*A*_1_	Weight factor for susceptible individuals	0.091	[31]
*A*_2_	Weight factor for exposed individuals	0.01	[31]
*A*_3_	Weight factor for infected individuals	0.04	[31]
*A*_4_	Weight factor for carrier individuals	0.05	[31]
*B*_1_	Weight factor for the controls *u*_1_	1.5	[31]
*B*_2_	Weight factor for the controls *u*_2_	2.7	[31]
*S*_0_	Susceptible individuals	0.493	[4]
*E*_0_	Exposed individuals	0.0035	[4]
*I*_0_	Acute infection individuals	0.0035	[4]
*C*_0_	Chronic HBV carriers	0.007	[4]
